# The effect of physical activity on smartphone addiction among Chinese college students: a chain mediation of self-control and health-promoting lifestyle

**DOI:** 10.3389/fpsyg.2026.1990964

**Published:** 2026-10-19

**Authors:** Hongsong Fu, Shirui Wang

**Affiliations:** 1 Hainan Police College, Haikou, Hainan, China; 2 Hainan Vocational College of Political Science and Law, Haikou, Hainan, China

**Keywords:** college students, health-promoting lifestyle, physical activity, self-control, smartphone addiction

## Abstract

**Objective:**

To explore the mechanism through which physical activity influences smartphone addiction tendency among Chinese college students, and to examine the chain mediating roles of self-control and health-promoting lifestyle.

**Methods:**

A questionnaire survey was conducted among 712 Chinese college students using the Physical Activity Rating Scale, the Self-Control Scale, the Health-Promoting Lifestyle Profile, and the Smartphone Addiction Tendency Scale. Structural equation modeling was employed for path analysis, and the bootstrap method was used to test the mediating effects.

**Results:**

The structural equation model showed a good fit. The direct effect of physical activity on smartphone addiction tendency was significant (*β* = −0.249, 95% *CI* [−0.348, −0.147]), accounting for 61.03% of the total effect. The total indirect effect was also significant (*β* = −0.159, 95% *CI* [−0.217, −0.102]), accounting for 38.97% of the total effect. This indirect effect comprised three pathways: the separate mediation of self-control (*β* = −0.090, accounting for 22.06%), the separate mediation of health-promoting lifestyle (*β* = −0.033, accounting for 8.09%), and the chain mediation of self-control → health-promoting lifestyle (*β* = −0.036, accounting for 8.82%, 95% *CI* [−0.060, −0.014]). The model explained 30.3%, 33.3%, and 21.9% of the variance in self-control, health-promoting lifestyle, and smartphone addiction tendency, respectively.

**Conclusion:**

Physical activity was significantly and negatively associated with smartphone addiction tendency among university students, with self-control and health-promoting lifestyle acting as sequential mediators. These findings suggest that interventions could integrate physical activity, self-control training, and health-promoting lifestyle education, with priority given to enhancing self-control to promote a health-promoting lifestyle, which may in turn be associated with lower smartphone addiction tendency. However, because of the cross-sectional design, causal relationships cannot be established; therefore, these intervention implications require validation in longitudinal or experimental studies.

## 1 Introduction

Since the launch of the first iPhone in 2007, smartphones have evolved from luxury items to indispensable tools in modern life. According to statistics from the China Internet Network Information Center, as of December 2025, the number of mobile internet users in China reached 1.125 billion, with 99.6% of internet users accessing the internet via mobile phones (CNNIC, 2026). While smartphones offer considerable convenience, addiction problems caused by their overuse are increasingly becoming a global public health challenge. University students are in the stage of “emerging adulthood,” transitioning from adolescence to adulthood, with self-regulatory abilities that are not yet fully mature, making them particularly vulnerable to smartphone dependence. Epidemiological surveys indicate that the detection rate of smartphone addiction tendency among Chinese university students is relatively high, and those who participate in physical activity show a relatively lower tendency toward smartphone addiction (Lai et al., 2025). Smartphone addiction tendency is closely associated with decreased sleep quality, increased risk of depression and anxiety, and impaired interpersonal relationships, making it a pressing real-world issue. Against this background, physical activity, as a malleable health behavior, has attracted widespread attention for its association with smartphone addiction tendency. Cross-sectional studies suggest that physical activity is significantly negatively associated with smartphone addiction tendency (Lai et al., 2025). However, the underlying psychological mechanisms linking physical activity and smartphone addiction tendency remain to be further elucidated.

Grounded in Self-Determination Theory and the Strength Model of Self-Control, the present study proposes that physical activity is associated with smartphone addiction tendency through a sequential mediation pathway involving self-control and health-promoting lifestyle. Unlike previous parallel mediation studies, to our knowledge, this study is the first to incorporate health-promoting lifestyle as a holistic construct into a sequential mediation model, examining a sequential mediating pathway from “capacity training” to “behavior internalization,” with the aim of providing theoretical hypotheses for intervention target selection. It should be noted that, given the cross-sectional design of the present study, the above pathways should be interpreted as a theoretically specified pattern of associations rather than a causal or longitudinal transmission mechanism.

## 2 Theoretical basis and hypothesis development

### 2.1 Theoretical basis: the strength model of self-control

This study adopts the Strength Model of Self-Control proposed by Baumeister et al. (2007) as its core explanatory framework. This model analogizes self-control to muscle strength: it can be strengthened through regular training, yet it can also be temporarily depleted by sustained exertion (Baumeister et al., 1998; Baumeister et al., 2007). Specifically, self-control relies on limited psychological resources; when individuals perform tasks that require willpower (e.g., resisting temptations, adhering to plans), these resources are consumed, leading to a subsequent decline in self-control performance (i.e., the ego depletion effect) (Baumeister et al., 1998). However, long-term and regular engagement in behaviors that demand self-discipline can, much like muscle training, raise the ceiling of one’s self-control resource reservoir (Baumeister et al., 2007; Muraven and Baumeister, 2000; Muraven et al., 1999).

In this study, physical activity is regarded as a behavior that requires sustained volitional effort and self-discipline. Its repeated execution constitutes effective training for prefrontal executive functions—particularly inhibitory control—thereby enhancing an individual’s general self-control resource reserves (Audiffren and André, 2015; Boat and Cooper, 2019; Englert, 2016). This theoretical framework provides a fundamental explanatory pathway for understanding the chain transmission mechanism of “physical activity → self-control → health-promoting lifestyle → smartphone addiction,” namely: by reinforcing self-control capacity, physical exercise enables individuals to more effectively mobilize cognitive inhibitory functions when facing immediate temptations of smartphone use, and simultaneously enhances their ability to consolidate discrete health behaviors into a systematic lifestyle, thereby doubly weakening the maintaining conditions of addictive behaviors.

### 2.2 The direct relationship between physical activity and smartphone addiction

Smartphone addiction tendency has become an increasingly serious behavioral problem among university students, and has been associated with declined academic performance, impaired sleep quality, and mental health problems (Li et al., 2020; Long et al., 2016). Physical activity, as an important component of a healthy lifestyle, has been suggested to be associated with lower smartphone addiction tendency among university students and may operate through multiple psychological pathways, including enhancing self-control (Yang et al., 2021; Zhong et al., 2020), alleviating psychological distress (Li et al., 2023), and reducing trait boredom (Qi et al., 2025).

A large body of cross-sectional and longitudinal studies has consistently shown that physical activity is significantly and negatively associated with smartphone addiction tendency (Guo et al., 2022; Yang et al., 2021; Zhong et al., 2020), and this association appears to be cross-contextually consistent across different cultural backgrounds and age groups (Xiao et al., 2022). However, these potential mechanisms operate at different explanatory levels and warrant differentiation. The behavioral substitution mechanism emphasizes that physical activity occupies limited leisure time and may be related to reduced time resources for smartphone use (Kushlev and Leitao, 2020), but it is more of a contextual opportunity mechanism and may not directly alter the psychological processes underlying addiction. The neurophysiological mechanism focuses on the effects of physical activity on dopaminergic signaling and neuroplasticity of the brain reward system (Cassilhas et al., 2016; Feng et al., 2019), which represents a distal biological foundation and is difficult to directly measure through self-report. The emotional regulation mechanism suggests that physical activity may promote positive emotional experiences and psychosocial adjustment, thereby potentially reducing the tendency to seek emotional compensation through smartphones (Grasdalsmoen et al., 2020; Morgan, 1985); however, emotional regulation itself is closely related to self-control and may partly represent a proximal outcome of self-control. The self-control mechanism, in contrast, draws on the Strength Model of Self-Control. Meta-analytic structural equation modeling suggests that physical activity is associated with an enhanced ability to inhibit immediate impulses, which in turn is associated with a lower risk of smartphone addiction (Lin et al., 2025). Intervention studies also indicate that structured exercise interventions lasting 12 to 16 weeks are associated with reduced smartphone addiction levels among university students (Xiao et al., 2021; Yang and Zeng, 2017).

Among these mechanisms, the present study focuses specifically on the self-control pathway for the following reasons. First, from the perspective of theoretical integration, Self-Determination Theory provides the motivational foundation: physical activity may satisfy the basic psychological needs for autonomy, competence, and relatedness, thereby promoting the internalization of health behaviors. The Strength Model of Self-Control then provides the self-regulatory mechanism, explaining how self-control, as a trainable capacity, supports cross-situational behavioral regulation. Second, from the perspective of variable relationships, self-control is a key psychological mechanism linking physical activity to health-promoting lifestyle. Health-promoting lifestyle is not a single behavior but a holistic pattern encompassing multiple dimensions such as nutrition, physical activity, stress management, health responsibility, interpersonal relationships, and self-actualization. Its formation and maintenance require strong cross-situational self-regulatory capacity. Third, from an intervention perspective, compared with neurophysiological changes, self-control is more malleable and can serve as a direct intervention target. Fourth, from the perspective of model logic, the sequential pathway of “self-control → health-promoting lifestyle → smartphone addiction tendency” reflects a sequential process from “capacity training” to “behavior internalization,” which helps explain how physical activity is associated with a broader pattern of health behaviors and, in turn, with lower smartphone addiction tendency. Behavioral substitution, neurophysiological, and emotional regulation mechanisms may serve as complementary explanations but are not the core pathways tested in this study. It should be noted that, given the cross-sectional design of the present study, the above pathways should be interpreted as a theoretically specified pattern of associations rather than a causal or longitudinal transmission mechanism.

### 2.3 The mediating role of self-control

Self-control refers to the ability of individuals to overcome impulses, habits, or automatic responses, and to consciously regulate their own behavior in accordance with social norms and long-term goals (Baumeister et al., 2007; Tan and Guo, 2008). It plays a critical role in the formation and maintenance of behavioral addictions (Brand et al., 2016). The Strength Model proposed by Baumeister et al. (2007) analogizes self-control to a muscle that can be strengthened through regular training but can also be temporarily depleted by sustained use (Baumeister et al., 1998). This theory provides a direct theoretical basis for understanding how physical activity influences smartphone addiction through self-control. It should be clarified that the self-control measured in this study operationally refers primarily to the individual’s capacity to mobilize prefrontal-driven inhibitory control when facing immediate temptations (e.g., smartphone use) in order to sustain long-term goals, which is highly congruent with the executive-function construct in the Strength Model (Hare et al., 2009; Hofmann et al., 2009). Neuroimaging studies have shown that inhibitory control, which is closely related to self-control, relies mainly on the activation of the prefrontal cortex (especially the dorsolateral prefrontal cortex) (Angius et al., 2019; Gong et al., 2025; Xiong et al., 2025), and regular physical activity has been demonstrated to effectively improve the structural and functional efficiency of this brain region (Colcombe et al., 2006; Erickson et al., 2011; Hu et al., 2021; Voss et al., 2013). Therefore, within the theoretical framework of this study, self-control is operationalized as a trait-like executive capacity that can be enhanced through behavioral training (Muraven and Baumeister, 2000), rather than as a purely dispositional tendency or motivational state.

A substantial body of empirical research confirms a significant positive correlation between physical activity and self-control. A survey by Yang et al. (2019) on 608 college students showed that physical activity was significantly positively correlated with self-control (*r* = 0.164, *p* < 0.001) and significantly positively predicted self-control levels (*β* = 0.168, *p* < 0.001). Zhong et al. (2020) found that individuals with high exercise volume exhibited significantly better self-control than those with low exercise volume. Neuroimaging studies indicate that acute mild exercise can activate the left dorsolateral prefrontal cortex and the frontopolar region, and the activation of these brain areas is closely associated with enhanced executive functions, particularly inhibitory control (Byun et al., 2014). Intervention studies have also confirmed that long-term regular aerobic exercise and group physical activities are positively associated with enhanced individual inhibitory control (Davis et al., 2011).

Meanwhile, the negative association between self-control and smartphone addiction has been extensively validated. Yang et al. (2019) found that self-control was significantly negatively correlated with mobile phone dependence (*r* = −0.563, *p* < 0.001) and significantly negatively predicted mobile phone dependence (*β* = −0.552, *p* < 0.001). Individuals with low self-control find it difficult to resist immediate temptations and are more prone to seeking short-term gratification, thereby increasing the risk of excessive smartphone use (Jiang and Zhao, 2016). Hofmann et al. (2009), drawing on the dual-system model, proposed that self-control failure arises from an imbalanced competition between the impulsive system and the reflective system over behavioral schemas. The effective inhibition of impulses by the reflective system relies on working memory capacity and self-control resources; when control resources are constrained by ego depletion, cognitive load, or trait-low self-control, the reflective system is impaired, and the impulsive system then dominates behavior (Hofmann et al., 2009).

Lin et al. (2025), using meta-analytic structural equation modeling that integrated 48 studies (75,541 participants), systematically evaluated the mediating effect of self-control between physical activity and smartphone addiction. Path analysis confirmed that self-control played a partial mediating role (indirect effect = −0.091, 95% *CI* [−0.118, −0.067]), with the mediating effect accounting for 49.7% of the total effect. Studies by Wang (2025) and Ye et al. (2025) also corroborated this mediating effect in college student samples. Therefore, this study proposes Hypothesis H2: Self-control mediates the relationship between physical activity and smartphone addiction among college students.

### 2.4 The mediating role of health-promoting lifestyle

Health-promoting lifestyle refers to the proactive and voluntary behavioral patterns that individuals adopt to maintain or enhance their health, encompassing multiple dimensions such as nutrition, physical activity, health responsibility, stress management, interpersonal relationships, and spiritual growth (Walker et al., 1987). There is a strong positive association between physical activity and health-promoting lifestyle. Regular physical activity not only directly improves individuals’ physical fitness and health literacy but also facilitates the formation and maintenance of other health behaviors through behavioral transfer effects (Xiang, 2013). According to Self-Determination Theory, physical exercise satisfies the three basic psychological needs of autonomy, competence, and relatedness, thereby stimulating intrinsic motivation and driving health behaviors to gradually internalize through external regulation, introjection, identification, and ultimately integrated regulation, resulting in a stable and enduring health-promoting lifestyle (Lai et al., 2025; Ryan and Deci, 2000).

A significant negative association exists between health-promoting lifestyle and smartphone addiction. As a positive daily activity system, a health-promoting lifestyle provides individuals with abundant behavioral alternatives, objectively reducing reliance on and time spent on smartphones (Liu and Sun, 2023). At the same time, by satisfying psychological needs and providing intrinsic fulfillment, a health-promoting lifestyle effectively reduces the motivation to seek immediate gratification and emotional compensation through smartphone use. The enhancement of self-control and the cultivation of a healthy lifestyle form a mutually reinforcing virtuous cycle (Baumeister et al., 2007). From the perspective of Compensatory Internet Use Theory, when individuals can obtain sufficient satisfaction from real-life healthy lifestyles, they have no need to turn to the virtual world for compensatory fulfillment (Kardefelt-Winther, 2014). Therefore, this study proposes Hypothesis H3: Health-promoting lifestyle mediates the relationship between physical activity and smartphone addiction among college students.

### 2.5 The chain mediating role

Physical activity, in addition to being directly negatively associated with smartphone addiction tendency, may also exert mediating effects through self-control and health-promoting lifestyle. These two mediators are not isolated from each other: self-control may be an important prerequisite for the formation of a health-promoting lifestyle, and together they may constitute a sequential pathway of “self-control → health-promoting lifestyle”.

Theoretically, the Strength Model of Self-Control proposed by Baumeister et al. (2007) emphasizes that self-control relies on limited psychological resources, can be enhanced through regular training, and mainly explains the capacity to inhibit impulses; however, the internalization of health behaviors requires supplementation by Self-Determination Theory (Ryan and Deci, 2000; Standage et al., 2012). According to Self-Determination Theory, the satisfaction of the needs for autonomy, competence, and relatedness facilitates the transformation of behavior from external constraints to intrinsic values. Within this framework, self-control is not only an inhibitory capacity but also a self-regulatory capacity that supports behavioral internalization: higher self-control enables individuals to persist with health behaviors even when motivation is not yet sufficient, and through repeated practice, to experience a sense of efficacy and value, thereby promoting the internalization of a healthy lifestyle. The integration of these two theories provides a more complete theoretical basis for the “self-control → health-promoting lifestyle” link.

Empirically, studies previously cited as evidence for sequential mediation (2023; 2024) mostly examined pathways such as “self-control → smartphone addiction” or “self-control → perceived stress” and cannot directly support the specific sequence of “self-control → health-promoting lifestyle” proposed in the present study. However, existing research provides direct support for this key link. The longitudinal study by Gilbert et al. (2022) showed that basic psychological need satisfaction predicted subsequent healthy lifestyle behaviors through self-control; recent research further supports the sequential mediation pathway of “self-control → health beliefs” (Hu et al., 2026). These findings provide an empirical basis for placing health-promoting lifestyle after self-control.

Brand et al. (2016)’s I-PACE model posits that predisposing factors can lead to addictive behaviors through affective and cognitive reactions and declined executive function. In the sequential pathway of the present study, physical activity may be associated with enhanced self-control, and improved self-control may enable individuals to reduce using smartphones to cope with stress, while a health-promoting lifestyle may provide alternative sources of satisfaction and positive behavioral choices. It should be noted that, given the cross-sectional design of the present study, the above pathways should be interpreted as a theoretically specified pattern of associations rather than a causal or longitudinal transmission mechanism. Accordingly, the present study proposes Hypothesis H4: Self-control and health-promoting lifestyle sequentially mediate the association between physical activity and smartphone addiction tendency among university students.

## 3 Research methods

### 3.1 Study design and participants

This study employed a cross-sectional survey design. A combination of convenience sampling and quota sampling was used to recruit full-time university students from eight universities in Hainan Province. Inclusion criteria were: (1) full-time university students; (2) owning a smartphone and being able to use it normally; and (3) providing voluntary informed consent. Exclusion criteria were: (1) part-time students (e.g., employed or correspondence students); and (2) students with severe physical or mental illness who were unable to complete the questionnaire independently. The sample size was determined based on the rule of thumb for structural equation modeling, which requires 10–20 cases per estimated parameter (Kline, 2016). Considering that the present study included 4 latent variables, 17 observed indicators, and a sequential mediation model, the minimum effective sample size was set at no less than 500.

Electronic questionnaires were developed and administered online via the Wenjuanxing platform. Before the formal survey, a pilot test was conducted with 50 university students with similar characteristics. Based on their feedback, the wording of items was slightly revised, and the average completion time was confirmed to be 15–20 min, after which the formal questionnaire was finalized. Formal data collection was conducted from March to April 2026. A total of 720 questionnaires were distributed via physical education teachers and class WeChat groups at universities in Hainan Province, China. A cover letter was placed on the first page of the questionnaire, explaining the purpose of the study, data use, voluntary participation, and anonymity. Clicking “agree and start” was considered as providing electronic informed consent.

Quality control measures included: (1) embedding attention check items; (2) making all items mandatory; (3) enabling IP restrictions to prevent duplicate submissions; and (4) randomizing the order of some scale items to reduce order effects. After questionnaire collection, two researchers independently reviewed and cleaned the data. Exclusion criteria were: (1) patterned responding; (2) missing response rate > 5%; and (3) failure to pass attention check items or completion time < 5 min. After cross-checking and discussion to reach consensus, 712 valid questionnaires were obtained, yielding a valid response rate of 98.9%. The demographic characteristics of the valid sample were as follows: 398 males (55.9%) and 314 females (44.1%); 392 freshmen (55.1%) and 320 sophomores (44.9%); 324 students (45.5%) from urban areas and 388 (54.5%) from rural areas.

It should be noted that the present sample was recruited from eight universities in Hainan Province and included only freshmen and sophomores, indicating clear regional and grade-level limitations in the sampling frame. Hainan has a tropical climate with high temperature and humidity throughout the year; therefore, the physical activity patterns of university students may differ systematically from those in temperate or cold regions. In addition, senior students face pressures such as employment and postgraduate entrance examinations, and their smartphone use patterns may differ fundamentally from those of lower-grade students. Consequently, the conclusions of the present study should be regarded as findings specific to “lower-grade university students in a tropical region” and should be generalized only cautiously to populations with characteristics similar to those of freshmen and sophomores at the eight universities in Hainan Province, rather than to the broader population of Chinese university students. Future studies should include multi-regional, multi-grade, and probability-based samples to examine the external validity of the present findings.

### 3.2 Measurement instruments

#### 3.2.1 Physical activity rating scale

This study adopted the Chinese version of the Physical Activity Rating Scale, which was revised by Liang (1994) based on the original scale developed by Hashimoto (1990). The scale was designed to assess participants’ physical activity volume over the past month. The items measured were: “What is the intensity of your physical exercise each time?”, “How long do you exercise each time?”, and “How often have you exercised in the past month?” Each dimension was rated on a 5-point Likert scale (1–5), corresponding to exercise intensity, exercise duration, and exercise frequency, respectively. The mean scores of the three dimensions were used as observed indicators for the latent variable of physical activity, with higher scores indicating higher levels of physical activity participation. In this study, the internal consistency reliability of the scale, as measured by Cronbach’s α, was 0.896.

#### 3.2.2 Smartphone addiction tendency scale for college students

This study adopted the Smartphone Addiction Tendency Scale for College Students developed by Xiong et al. (2012). The scale comprises four dimensions—withdrawal symptoms, salience, social comfort, and mood change—with a total of 16 items. All items were rated on a 5-point Likert scale ranging from 1 (“completely inconsistent”) to 5 (“completely consistent”), with higher scores indicating greater smartphone addiction tendency. Confirmatory factor analysis demonstrated a good model fit: *χ^2^*/*df* = 3.169, TLI = 0.967, CFI = 0.972, RMSEA = 0.055, and SRMR = 0.025. The overall Cronbach’s α coefficient of the scale was 0.957. In the present sample, the Cronbach’s α coefficients for the subscales ranged from 0.779 to 0.889.

#### 3.2.3 Self-control scale

This study adopted the Self-Control Scale revised by Tan and Guo (2008) to assess college students’ self-control ability. The scale comprises five dimensions—impulse control, healthy habits, resistance to temptation, focused work, and moderation in entertainment—with a total of 19 items. All items are rated on a 5-point Likert scale ranging from 1 (completely inconsistent) to 5 (completely consistent). The scale includes 15 reverse-scored items; thus, higher total scores indicate greater self-control ability. Confirmatory factor analysis demonstrated a good model fit: *χ^2^*/*df* = 2.464, TLI = 0.973, CFI = 0.976, RMSEA = 0.045, and SRMR = 0.023. In the present sample, the Cronbach’s α coefficients for the subscales ranged from 0.792 to 0.885.

#### 3.2.4 Health-promoting lifestyle profile

This study adopted the Chinese version of the Health-Promoting Lifestyle Profile-II Revised (HPLP-II R) developed by Cao et al. (2016), which was revised based on the original Health-Promoting Lifestyle Profile proposed by Walker et al. (1987). The scale comprises six dimensions—health responsibility, stress management, nutrition, interpersonal relationships, physical activity, and spiritual growth—with a total of 40 items. All items are rated on a 5-point Likert scale ranging from 1 (completely inconsistent) to 5 (completely consistent), with higher scores indicating higher levels of health-promoting lifestyle. Confirmatory factor analysis demonstrated a good model fit: *χ^2^*/*df* = 2.690, TLI = 0.916, CFI = 0.921, RMSEA = 0.049, and SRMR = 0.035. In the present sample, the Cronbach’s α coefficients for the subscales ranged from 0.825 to 0.915. Given that the “physical activity” dimension of the HPLP-II overlaps conceptually with the independent variable (physical activity), this dimension was excluded in a sensitivity analysis, and the model was re-tested. The results showed that the significance and direction of the core paths remained unchanged (chain mediation effect: *β* = −0.034, 95% *CI* [−0.057, −0.013]), indicating that this overlap did not substantially affect the robustness of the study findings.

### 3.3 Statistical analysis

Data analyses were performed using SPSS 26.0, AMOS 24.0, and Mplus 8.3. All statistical tests were two-tailed, and the significance level was set at α = 0.05.

#### 3.3.1 Common method bias test

As all variables were assessed through self-report measures, common method bias may pose a potential threat. This study employed Harman’s single-factor test to evaluate this bias: all measurement items were entered into SPSS 26.0 for unrotated principal component analysis. If the variance explained by the first principal component was below the critical threshold of 40% (Podsakoff et al., 2003a), common method bias was considered not to be a serious concern.

#### 3.3.2 Reliability and validity tests

Reliability and validity analyses were jointly conducted using SPSS 26.0 and AMOS 24.0. Reliability was assessed by calculating Cronbach’s α coefficients for each scale and its subdimensions using SPSS 26.0, with an α > 0.70 considered acceptable (Hu and Bentler, 1999). Validity was evaluated through confirmatory factor analysis (CFA) using AMOS 24.0 to examine the structural validity of the latent variables. Model fit was assessed using the following indices and criteria: *χ^2^*/*df* < 3 (ideal) or < 5 (acceptable); CFI > 0.95 (excellent) or > 0.90 (acceptable); TLI > 0.95 (excellent) or > 0.90 (acceptable); RMSEA < 0.06 (excellent) or < 0.08 (acceptable); and SRMR < 0.06 (excellent) or < 0.08 (acceptable) (Hu and Bentler, 1999). Convergent validity was evaluated using the average variance extracted (AVE), with AVE > 0.50 considered acceptable; composite reliability (CR) was considered acceptable at CR > 0.70. Discriminant validity was assessed using the Fornell-Larcker criterion, which requires that the square root of the AVE for each latent variable be greater than its correlations with other latent variables (Fornell and Larcker, 1981).

#### 3.3.3 Normality assessment and descriptive statistics

Normality was assessed using SPSS 26.0 by examining univariate normality of the observed indicators for each core variable. Skewness and kurtosis were calculated for each indicator. Following the criteria recommended by Kline (2023), absolute skewness values < 2 and absolute kurtosis values < 7 were considered indicative of approximate normal distribution. Descriptive statistics and correlation analyses were performed using the PROCESS macro for SPSS (version 3.5) (Hayes, 2017) to compute means (M), standard deviations (SD), and Pearson product–moment correlation coefficients among the core latent variables. The direction and significance of the correlation coefficients were examined to preliminarily assess the relationships among variables, thereby providing a prerequisite basis for subsequent mediation analyses.

#### 3.3.4 Structural equation modeling and mediation effect testing

This study used Mplus 8.3 to construct a structural equation model (SEM) to examine the path relationships among latent variables and the sequential mediation effects. Observed indicators were constructed as follows: for the unidimensional Physical Activity Scale, items were randomly parceled into three indicators; for the multidimensional constructs of self-control, health-promoting lifestyle, and smartphone addiction tendency, dimension mean scores were used as observed indicators (five dimensions for self-control, six for health-promoting lifestyle, and four for smartphone addiction tendency). The rationale for using dimension mean scores rather than random parceling was fourfold. First, these constructs are theoretically multidimensional, and dimension mean scores preserve their theoretical structure. Second, random parceling may obscure localized model misfit and blur the differential functions of dimensions. Third, the dimension mean scores showed acceptable reliability in the present sample. Fourth, using dimension mean scores as observed indicators yields a more parsimonious and interpretable model and is consistent with the measurement model presented in Table 1. The latent variables represented the common variance among the dimensions of each construct.

**Table 1:** Measurement model: confirmatory factor analysis results.

Latent variable	Indicator	Standardized factor loading (λ)	S. E.	C. R.	*p*	CR	AVE
PA	Exercise duration	0.821	0.011	77.848	***	0.889	0.728
	Exercise intensity	0.884	0.01	90.565	***		
	Exercise frequency	0.884	0.01	90.162	***		
SA	Withdrawal symptoms	0.949	0.007	139.408	***	0.958	0.852
	Salience	0.926	0.007	134.338	***		
	Social comfort	0.917	0.01	95.539	***		
	Mood change	0.910	0.01	91.614	***		
SC	Impulse control	0.935	0.008	119.688	***	0.96	0.828
	Healthy habits	0.904	0.011	83.063	***		
	Resistance to temptation	0.920	0.008	113.203	***		
	Focused work	0.905	0.009	96.649	***		
	Moderation in entertainment	0.892	0.011	84.612	***		
HPL	Interpersonal relationships	0.871	0.013	67.480	***	0.947	0.75
	Nutrition	0.889	0.010	92.747	***		
	Health responsibility	0.901	0.011	83.627	***		
	Physical activity	0.889	0.011	80.433	***		
	Stress management	0.850	0.013	64.369	***		
	Spiritual growth	0.803	0.019	42.007	***		

S.E. = standard error; C.R. = critical ratio; CR = composite reliability; AVE = average variance extracted. ***p < 0.001. All factor loadings were estimated using maximum likelihood (ML).

The model was specified as follows: physical activity (PA) predicted self-control (SC), health-promoting lifestyle (HPL), and smartphone addiction (SA); SC predicted HPL and SA; and HPL predicted SA. Gender, grade, and place of origin were included as covariates and regressed on all endogenous variables to control for confounding effects. Model fit was evaluated using the same indices and criteria as in the confirmatory factor analysis (*χ^2^/df*, CFI, TLI, RMSEA, SRMR). Mediation effects were tested using the bias-corrected percentile bootstrap method with 5,000 resamples to compute 95% confidence intervals for each indirect effect; an indirect effect was considered significant if the confidence interval did not include zero (Preacher and Hayes, 2008). Standardized effect sizes for each path and R^2^ values for endogenous variables were also reported to evaluate the overall explanatory power of the model.

In addition, to enhance the robustness of the findings, SPSS PROCESS macro (Preacher and Hayes, 2008) was used to conduct a sequential mediation analysis based on observed variables (dimension mean scores) as a robustness analysis of the Mplus latent variable model, rather than as cross-validation. Gender, grade, and place of origin were included as covariates in the PROCESS analysis, and the significance of indirect effects was tested using the bootstrap method (5,000 resamples).

## 4 Results

### 4.1 Common method Bias test and normality assessment

Common method bias was first examined using Harman’s single-factor test via principal component analysis as an initial diagnostic. To further evaluate the potential impact of common method bias, an unmeasured latent method factor approach was employed (Podsakoff et al., 2003b). Building on the four-factor measurement model, all observed indicators were simultaneously loaded onto their respective theoretical constructs and a common method latent factor. After adding the common method factor, the changes in model fit were as follows: CFI = 0.002, TLI = 0.001, and RMSEA = 0.001. All improvements were well below the 0.01 threshold (Podsakoff et al., 2003b), suggesting that common method bias was unlikely to pose a serious threat to the interpretation of the results. Without rotation, seven factors had eigenvalues greater than 1, and the first common factor accounted for 31.97% of the variance, which was below the 40% threshold, further indicating that common method bias was not substantial and that subsequent analyses could proceed. Data normality was assessed using the criteria recommended by Kline (2023): absolute skewness < 2 and absolute kurtosis < 7. For the four core latent variables, absolute skewness ranged from 0.107 to 0.486, and absolute kurtosis ranged from 0.287 to 0.515, both below the threshold values (see Table 2), indicating that the data did not show serious departures from univariate normality and were suitable for maximum likelihood estimation. The normality results for all 18 observed indicators are provided in Supplementary Table S1. It should be noted that the above analyses were based on cross-sectional data, and the results should be interpreted as patterns of associations among variables rather than as evidence of causal or temporal ordering.

**Table 2:** Univariate normality test results for core latent variables (*N* = 712).

Latent variable	Skewness	S.E. of skewness	Kurtosis	S.E. of kurtosis
PA	−0.486	0.092	−0.515	0.183
SA	0.107	0.092	−0.287	0.183
SC	−0.321	0.092	−0.339	0.183
HPL	−0.203	0.092	−0.338	0.183

S.E. = standard error. All absolute skewness values are < 2 and absolute kurtosis values are < 7, indicating approximate univariate normality according to Kline’s (2023) criteria.

### 4.2 Descriptive statistics and correlation analysis

The means, standard deviations, and Pearson correlation coefficients of the core latent variables are presented in Table 3. The results showed that physical activity was significantly and negatively correlated with smartphone addiction (*r* = −0.469, *p* < 0.001), and significantly and positively correlated with self-control (*r* = 0.550, *p* < 0.001) and health-promoting lifestyle (*r* = 0.469, *p* < 0.001). Self-control was significantly and positively correlated with health-promoting lifestyle (*r* = 0.577, *p* < 0.001), and both were significantly and negatively correlated with smartphone addiction (*r* = −0.540 and −0.468, both *p* < 0.001). The directions and magnitudes of these correlations were consistent with theoretical expectations, providing an initial basis of associations for the subsequent mediation model testing. It should be noted that these results reflect only cross-sectional associations among variables and cannot establish temporal ordering or causality. Furthermore, significant bivariate correlations are not a necessary prerequisite for mediation analysis. The full correlation matrix including all 18 observed indicators is provided in Supplementary Table 2.

**Table 3:** Pearson correlations among core latent variables (*N* = 712).

Latent Variable	M	SD	PA	SA	SC	HPL
PA	3.52	1.08	—			
SA	3.02	0.72	−0.469***	—		
SC	3.64	0.69	0.550***	−0.540***	—	
HPL	3.61	0.61	0.469***	−0.468***	0.577***	—

*** *p* < 0.001 (two‑tailed). All correlations were calculated based on the mean scores of the latent variable dimensions.

### 4.3 Measurement model testing

Before testing the structural model, the measurement model was first evaluated using confirmatory factor analysis (CFA) to examine the relationships between each latent variable and its observed indicators. Maximum likelihood (ML) estimation was used to estimate the parameters of the measurement model. As shown in Table 4, the measurement model fitted the data well: *χ*^2^(129) = 266.20, *χ^2^/df* = 2.064, CFI = 0.990, TLI = 0.988, RMSEA = 0.039 (90% *CI* [0.032, 0.045]), and SRMR = 0.019. Overall, all fit indices reached excellent levels, supporting subsequent structural model analysis; however, the subsequent paths should be interpreted as associations among variables rather than causal effects.

**Table 4:** Model fit indices.

Index	Value	Criterion	Evaluation
*χ^2^*(*df*)	266.20 (129)	-	-
*χ^2^*/*df*	2.064	< 3.0 excellent; < 5.0 acceptable	Excellent
TLI	0.988	> 0.95 excellent; > 0.90 acceptable	Excellent
CFI	0.990	> 0.95 excellent; > 0.90 acceptable	Excellent
RMSEA	0.039	< 0.06 excellent; < 0.08 acceptable	Excellent
SRMR	0.019	< 0.06 excellent; < 0.08 acceptable	Excellent

The standardized factor loadings of the observed indicators ranged from 0.803 to 0.949, all of which were statistically significant (*p* < 0.001) (see Table 1), indicating that the observed indicators adequately represented their respective latent variables. The composite reliability (CR) of the latent variables ranged from 0.889 to 0.960, and the average variance extracted (AVE) ranged from 0.728 to 0.852, both exceeding the recommended thresholds (CR > 0.70, AVE > 0.50), indicating good reliability and convergent validity of the scales.

Discriminant validity was examined using the Fornell-Larcker criterion: the square root of the AVE for each latent variable should be greater than its correlations with other latent variables. The square roots of the AVE for the four latent variables were all greater than their correlations with other latent variables, indicating good discriminant validity, and the four constructs were conceptually independent and distinguishable. As shown in Table 5, the square roots of the AVE for each latent variable (0.853–0.923) were all greater than the absolute values of their correlations with other latent variables (0.468–0.577), indicating good discriminant validity among the four latent variables.

**Table 5:** Discriminant validity test.

Variable	AVE	PA	SA	SC	HPL
PA	0.728	**0.853**			
SA	0.852	−0.469	**0.923**		
SC	0.828	0.55	−0.54	**0.91**	
HPL	0.750	0.469	−0.468	0.577	**0.866**

Boldface values on the diagonal are the square roots of the average variance extracted (AVE); off‑diagonal values are the correlations between latent variables. All correlations are significant at *p* < 0.001 (two‑tailed).

In summary, the reliability, convergent validity, and discriminant validity of the measurement model all reached satisfactory levels, supporting subsequent structural model and mediation analyses. It should be noted that, given the cross-sectional design of the present study, the path coefficients in the subsequent structural model should be interpreted as patterns of associations among variables rather than as evidence of causal or temporal ordering.

### 4.4 Structural model and mediation effect testing

To enhance the robustness of the findings, both the SPSS PROCESS macro and Mplus 8.3 were used to conduct the sequential mediation analysis as complementary robustness analyses rather than cross-validation. The PROCESS analysis was based on observed variables (dimension mean scores), with gender, grade, and place of origin included as covariates. The regression results (see Table 6) indicated that, after controlling for demographic variables, physical activity was significantly and positively associated with self-control (*β* = 0.336, *p* < 0.001) and health-promoting lifestyle (*β* = 0.109, *p* < 0.001), and significantly and negatively associated with smartphone addiction (*β* = −0.156, *p* < 0.001); self-control was significantly and positively associated with health-promoting lifestyle (*β* = 0.363, *p* < 0.001) and significantly and negatively associated with smartphone addiction (*β* = −0.179, *p* < 0.001); and health-promoting lifestyle was significantly and negatively associated with smartphone addiction (*β* = −0.182, *p* < 0.001). None of the demographic covariates showed statistically significant paths to the core variables.

**Table 6:** Regression coefficients for the mediation model (*N* = 712).

Outcome variable	Predictor variable	*β*	S. E.	*t*	*p*	95% CI	*R^2^*
SC	PA	0.336	0.021	15.914	<0.001	[0.295, 0.378]	0.265
	Grade	−0.028	0.044	−0.648	0.517	[−0.114, 0.057]	
	Gender	−0.043	0.044	−0.981	0.327	[−0.129, 0.043]	
	Residence	−0.011	0.044	−0.24	0.81	[−0.096, 0.075]	
HPL	PA	0.109	0.021	5.315	<0.001	[0.069, 0.150]	0.309
	SC	0.363	0.031	11.567	<0.001	[0.302, 0.425]	
	Grade	−0.03	0.036	−0.817	0.415	[−0.101, 0.042]	
	Gender	0.02	0.037	0.55	0.583	[−0.052, 0.092]	
	Residence	0.063	0.037	1.737	0.083	[−0.008, 0.135]	
SA	PA	−0.156	0.027	−5.714	<0.001	[−0.210, −0.102]	0.206
	SC	−0.179	0.045	−4.001	<0.001	[−0.266, −0.091]	
	HPL	−0.182	0.049	−3.705	<0.001	[−0.278, −0.085]	
	Grade	0.056	0.047	1.188	0.235	[−0.037, 0.149]	
	Gender	−0.014	0.048	−0.294	0.769	[−0.108, 0.080]	
	Residence	−0.047	0.048	−0.986	0.325	[−0.141, 0.047]	

PA = Physical Activity; SC = Self‑Control; HPL = Health‑Promoting Lifestyle; SA = Smartphone Addiction. All reported coefficients are unstandardized regression coefficients. Covariates: Grade (1 = freshman, 2 = sophomore), Gender (1 = male, 2 = female), Residence (1 = urban, 2 = rural).

Building on the validated measurement model, a structural equation model was further constructed to examine the direct path between physical activity and smartphone addiction, as well as the sequential indirect paths through self-control and health-promoting lifestyle. The structural equation model results indicated that all six direct path hypotheses were supported, and all three indirect paths (including the sequential mediation path) were significant. The model conformed to a partial mediation pattern, with the direct path accounting for 61.03% of the total association and the total indirect path accounting for 38.97%. The bootstrap 95% confidence interval for the sequential mediation path did not include zero, supporting the statistical significance of the indirect association pathway of “physical activity → enhanced self-control → promoted health-promoting lifestyle → reduced smartphone addiction”.

The structural model results are presented in Table 7. All six direct paths reached statistical significance (*p* < 0.01). Specifically, physical activity was significantly and positively associated with self-control (*β* = 0.550, *p* < 0.001) and health-promoting lifestyle (*β* = 0.215, *p* < 0.001), and significantly and negatively associated with smartphone addiction (*β* = −0.249, *p* < 0.001); self-control was significantly and positively associated with health-promoting lifestyle (*β* = 0.430, *p* < 0.001) and significantly and negatively associated with smartphone addiction (*β* = −0.163, *p* < 0.001); and health-promoting lifestyle was significantly and negatively associated with smartphone addiction (*β* = −0.154, *p* < 0.001). The model explained 21.9% of the variance in smartphone addiction, 30.3% in self-control, and 33.3% in health-promoting lifestyle.

**Table 7:** Path coefficients for the structural model.

Path	Std. (*β*)	S.E.	C.R.	*P*	95% bootstrap CI	R^2^
PA → SC	0.55	0.031	17.841	<0.001	[0.487, 0.610]	0.303
SC → HPL	0.43	0.041	10.378	<0.001	[0.347, 0.510]	0.333
PA → HPL	0.215	0.041	5.258	<0.001	[0.136, 0.295]	
PA → SA	−0.249	0.051	−4.920	<0.001	[−0.348, −0.117]	0.219
HPL → SA	−0.154	0.044	−3.479	0.001	[−0.239, −0.065]	
SC → SA	−0.163	0.046	−3.526	<0.001	[−0.251, −0.070]	

Std. (β) = standardized regression coefficient; S.E. = standard error; C.R. = critical ratio; CI = confidence interval; R^2^ = coefficient of determination (variance explained). Bootstrap CIs were estimated using 5,000 resamples. All *p*‑values are two‑tailed.

Mediation effects were tested using the bootstrap method (5,000 resamples). As shown in Table 8 and Figure 1, the bootstrap 95% confidence intervals for all three indirect paths did not include zero: indirect path 1 (physical activity → self-control → smartphone addiction) was −0.090 (22.06% of the total association, *CI* [−0.142, −0.036]); indirect path 2 (physical activity → health-promoting lifestyle → smartphone addiction) was −0.033 (8.09% of the total association, *CI* [−0.058, −0.014]); and the sequential mediation path (physical activity → self-control → health-promoting lifestyle → smartphone addiction) was −0.036 (8.82% of the total association, *CI* [−0.060, −0.014]), supporting the statistical significance of this sequential indirect path. The total indirect path was −0.159 (38.97% of the total association, *CI* [−0.217, −0.102]). It should be noted that the percentages of the total association should be interpreted cautiously and should not be given substantive meaning beyond what the cross-sectional design can support.

**Table 8:** Mediation effect tests.

Effect	Path	Standardized indirect effect	S. E.	95% bootstrap CI	*p*	Percentage of total effect
Indirect effects	PA → SC → SA	−0.09	0.027	[−0.142, −0.036]	0.001	22.06%
	PA → HPL → SA	−0.033	0.012	[−0.058, −0.014]	0.004	8.09%
	PA → SC → HPL → SA	−0.036	0.012	[−0.060, −0.014]	0.001	8.82%
Total indirect effect		−0.159	0.03	[−0.217, −0.102]	<0.001	38.97%
Direct effect	Direct path	−0.249	0.051	[−0.348, −0.147]	<0.001	61.03%
Total effect		−0.408	0.046	[−0.499, −0.319]	<0.001	100.00%

**Figure 1:**
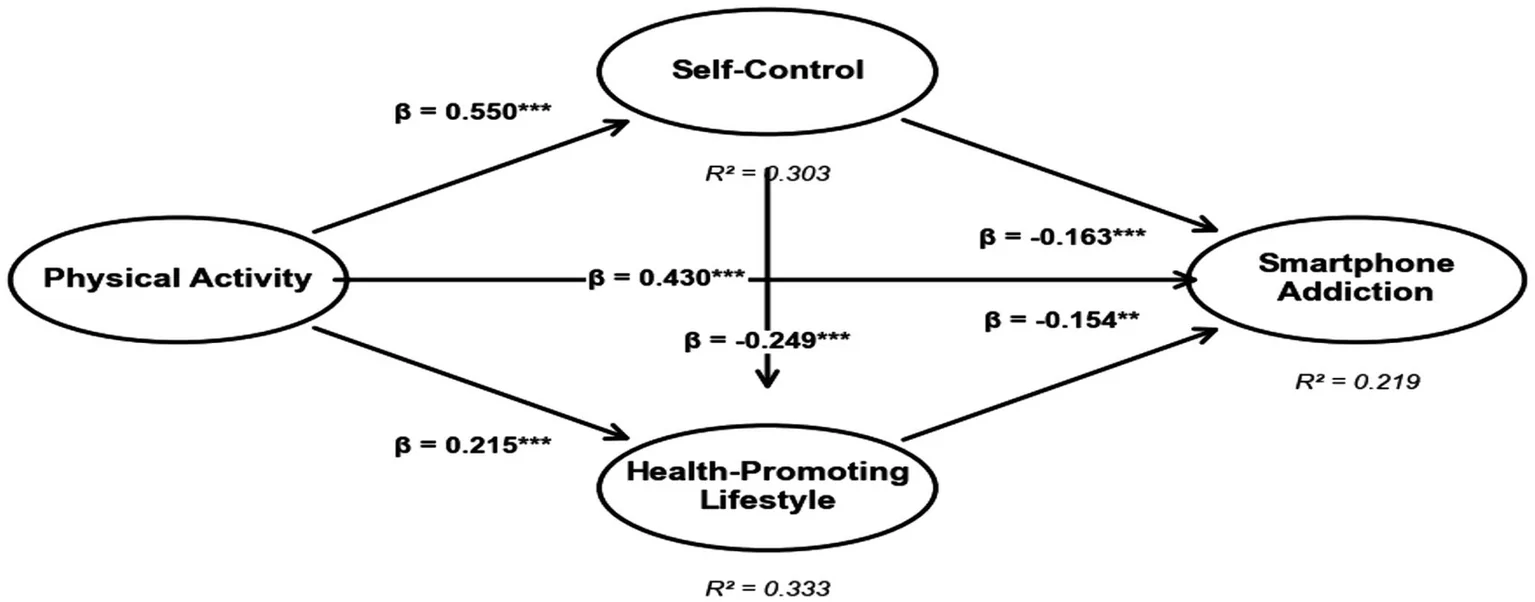
Structural equation modeling path diagram with standardized path coefficients.

In summary, the pattern of associations between physical activity and smartphone addiction was consistent with a partial mediation model—characterized by both a significant direct negative association (61.03% of the total association) and a significant indirect association through the sequential path of “enhanced self-control → promoted health-promoting lifestyle.” Given the cross-sectional design of the present study, these results should be interpreted as patterns of associations among variables rather than as evidence of causal or temporal ordering.

## 5 Discussion

### 5.1 The direct relationship between physical activity and smartphone addiction

The present study found that physical activity was significantly and directly negatively associated with smartphone addiction among university students (*β* = −0.249, 95% *CI* [−0.348, −0.147]), with the direct path accounting for 61.03% of the total association, consistent with Hypothesis H1. This result is consistent with previous empirical findings in China and abroad (Ke et al., 2024; Lai et al., 2025; Xiao et al., 2022). At the cross-sectional level, Lai et al. (2025) surveyed 3,506 university students in western China and found that the prevalence of smartphone addiction was lower among those who participated in physical activity than among those who did not (*OR* = 0.70, 95% *CI*: 0.59–0.82). Ke et al. (2024) further found that physical activity was significantly and negatively correlated with smartphone addiction (*r* = −0.124, *p* < 0.01). Systematic reviews and meta-analyses provide higher-level evidence for this association: a meta-analysis of 29 studies reported a moderate, significant negative correlation between the two (*r* = −0.349, *p* < 0.001) (Yu et al., 2025). Longitudinal and intervention studies also suggest that physical activity is associated with lower subsequent levels of smartphone addiction and that exercise interventions are associated with reductions in addiction levels (Thapliyal et al., 2025; Wang et al., 2024). The cross-study consistency of effect sizes suggests that the negative association between physical activity and smartphone addiction among university students is relatively robust. Notably, the total association observed in the present study (*β* = −0.408) was larger than the pooled effect size reported by Xiao et al. (2022) (*r* = −0.349). This difference may be partly attributable to the correction for measurement error in latent variable structural equation modeling, and may also be influenced by regional characteristics, sample composition, and other factors. However, because the compared statistics were derived from different analytical frameworks and samples, this difference should be interpreted cautiously and not overinterpreted. Future studies could further examine the moderating roles of regional, climatic, and measurement-related factors in this association.

Possible explanations for the negative association between physical activity and smartphone addiction can be understood through the following pathways; however, these mechanisms were not directly tested in the present study and should be regarded as possible explanations rather than established mechanisms. First, the behavioral substitution mechanism: physical activity occupies individuals’ leisure time and reduces the time window for smartphone use, thereby potentially lowering the cumulative risk of overuse (Grimaldi-Puyana et al., 2020). Second, the neurophysiological mechanism: physical activity may enhance dopaminergic signaling and modulate neuroplasticity of the brain reward system (Cassilhas et al., 2016), potentially replacing the immediate gratification from smartphones with healthier sources of reward and weakening the reinforcement cycle of addictive behavior. Third, the emotional regulation pathway: physical activity may alleviate negative emotions such as anxiety, depression, and stress, thereby reducing the motivation to seek emotional compensation through smartphones (Grasdalsmoen et al., 2020). In addition, physical activity may improve prefrontal executive function, particularly inhibitory control, thereby helping individuals engage cognitive control when confronted with the immediate temptation of smartphone use and inhibit impulses toward overuse (Byun et al., 2014). It should be emphasized that the above neurobiological and executive function explanations are speculative mechanisms that require direct testing in future research.

In summary, the present study found a negative association between physical activity and smartphone addiction among university students (with the direct path accounting for 61.03% of the total association), providing preliminary practical implications for university physical education and student affairs: increasing university students’ participation in physical activity may be a promising intervention target for smartphone addiction. However, the effect sizes of the independent mediation through health-promoting lifestyle and the sequential mediation were relatively small, and their practical significance should be interpreted cautiously. In addition, the present findings are consistent with some assumptions of frameworks such as I-PACE, but they represent a preliminary theoretical integration and cannot establish causal mechanisms or intervention efficacy. Future longitudinal and experimental studies are still needed for validation.

### 5.2 The mediating role of self-control

The present study found that the indirect path through self-control between physical activity and smartphone addiction among university students was significant (*β* = −0.090, 95% *CI* [−0.142, −0.036]), accounting for 22.06% of the total association, consistent with Hypothesis H2. This finding suggests that physical activity may be associated with lower smartphone addiction tendency through the indirect pathway of self-control, and this finding is consistent with previous empirical findings (Lin et al., 2025; Wang et al., 2024; Yang et al., 2019; Ye et al., 2025). Yang et al. (2019) surveyed 608 university students and found a significant indirect association through self-control between physical activity and mobile phone dependence. The cross-lagged study by Wang et al. (2024) further suggested that physical activity was positively associated with subsequent levels of self-control (*β* = 0.431, *p* < 0.001) and was indirectly associated with subsequent smartphone addiction through self-control (the indirect path accounted for 48.4% of the total association). In terms of effect size, the meta-analytic structural equation modeling study by Lin et al. (2025) (48 studies, 75,541 participants) reported an indirect effect of self-control of −0.091, accounting for 49.7% of the total association. Ye et al. (2025) also found a significant indirect association between physical activity and smartphone addiction through self-control (*β* = −0.444, *p* < 0.001).

The indirect role of self-control can be understood from the following theoretical perspectives, although these explanations are theoretical speculations and were not directly tested in the present study. First, the Strength Model of Self-Control (Baumeister et al., 2007). Physical activity, as a behavior requiring sustained volitional effort and self-discipline, may serve as effective training for self-control capacity. When individuals persist in completing exercise plans and overcome fatigue and inertia during exercise, their self-control resources may be exercised and expanded. This enhanced control capacity may then transfer to other behavioral domains, enabling individuals to better initiate cognitive control when facing the immediate temptation of smartphone use and to inhibit impulses toward overuse. Second, the dual-system model (Hofmann et al., 2009). Individuals with low self-control have difficulty resisting immediate temptations and are more likely to pursue immediate gratification, thereby increasing the risk of excessive smartphone use (Jiang and Zhao, 2016). Physical activity may enhance the control function of the reflective system, helping individuals make more adaptive choices between the immediate impulse to scroll on their phones and the long-term goal of restrained use. Third, neurophysiological mechanisms (Byun et al., 2014; Davis et al., 2011). Neuroimaging studies have shown that acute mild exercise can activate the left dorsolateral prefrontal cortex and frontal pole regions, and activation of these brain regions is closely associated with enhanced inhibitory control. Long-term regular aerobic exercise and team-based physical activity may also enhance individuals’ inhibitory control, providing a possible neurobiological basis for improvements in self-control. It should be emphasized that the above neurobiological explanations are speculative mechanisms that require direct testing in future research.

In summary, the present study found that the indirect role of self-control was consistent with a partial mediation pattern in the association between physical activity and smartphone addiction among university students (accounting for 22.06% of the total association). This finding suggests that physical activity may be a promising intervention target and may exert its effects by enhancing individuals’ self-regulatory capacity, providing testable intervention hypotheses for the prevention and intervention of smartphone addiction among university students. However, given the cross-sectional design of the present study, this indirect path cannot establish a causal mediation mechanism, and its practical significance should be interpreted cautiously. Future longitudinal and experimental studies are still needed for validation.

### 5.3 The mediating role of health-promoting lifestyle

The present study found that the indirect path through health-promoting lifestyle between physical activity and smartphone addiction among university students was significant (*β* = −0.033, 95% *CI* [−0.058, −0.014]), accounting for 8.09% of the total association, consistent with Hypothesis H3. This finding suggests that physical activity may be associated with lower smartphone addiction tendency through the promotion of a health-promoting lifestyle; however, the effect size of this indirect path was relatively small, and its practical significance should be interpreted cautiously.

This finding is directionally consistent with previous research. Ding et al. (2025) surveyed 1,002 university students and found that smartphone addiction was significantly and negatively correlated with health-promoting lifestyle (*r* = −0.074, *p* < 0.01). Xie et al. (2026) studied 2,317 university students and found that health-promoting lifestyle played a significant indirect role between smartphone addiction and sleep quality. In addition, a study of Korean university students suggested that smartphone addiction tendency may play an indirect role between perceived stress and health-promoting lifestyle (Won and Sin, 2018). These studies collectively suggest that health-promoting lifestyle may be related to mechanisms associated with smartphone addiction, but none can establish causality.

The indirect role of health-promoting lifestyle can be understood from the following theoretical perspectives, although these explanations are theoretical speculations and were not directly tested in the present study. First, the behavioral substitution mechanism. Health-promoting lifestyle encompasses multiple dimensions, including nutrition, physical activity, health responsibility, stress management, interpersonal relationships, and spiritual growth, and may provide individuals with abundant alternative behavioral choices, thereby reducing smartphone use time. Second, the psychological need satisfaction pathway. According to Self-Determination Theory (Xiang, 2013), health-promoting lifestyle may provide intrinsic satisfaction by fulfilling basic psychological needs for competence, autonomy, and relatedness. When individuals obtain sufficient psychological satisfaction through a healthy lifestyle in real life, they may be less likely to seek compensatory satisfaction in the virtual world, which is consistent with the logic of compensatory internet use theory (Kardefelt-Winther, 2014). Third, the behavioral transfer effect. Physical activity, as a core component of health-promoting lifestyle, may promote the formation and maintenance of other health behaviors through behavioral transfer. Improvements in self-control and the cultivation of a healthy lifestyle may form a mutually reinforcing cycle, which may be associated with a lower risk of smartphone addiction.

In summary, the present study found that the indirect role of health-promoting lifestyle in the association between physical activity and smartphone addiction among university students was consistent with a partial mediation pattern (accounting for 8.09% of the total association). However, the effect size was small, and its practical significance is limited. This finding provides only preliminary implications for university health education: in interventions targeting smartphone addiction through physical activity, it may be worth considering guiding university students to extend the positive effects of physical activity to broader health behavior domains. Nevertheless, this intervention approach requires validation in longitudinal and experimental studies.

### 5.4 Theoretical implications of the chain mediation effect

The core finding of the present study was that physical activity was associated with lower smartphone addiction tendency through the sequential pathway of “self-control → health-promoting lifestyle” (*β* = −0.036, 95% *CI* [−0.060, −0.014]), accounting for 8.82% of the total association, consistent with Hypothesis H4. This finding supports the presence of multiple association pathways between physical activity and smartphone addiction. To our knowledge, this study is the first to incorporate health-promoting lifestyle as a multidimensional holistic construct into a sequential mediation model, suggesting a possible sequential association between self-control and health-promoting lifestyle rather than treating them merely as parallel mediators. It should be noted that the effect size of this sequential indirect pathway was small, and its practical significance should be interpreted cautiously.

This sequential pathway is directionally consistent with previous findings. Wang (2025) found that physical activity was associated with smartphone addiction through the sequential pathway of “self-control → resilience” (sequential indirect effect = −0.0259, accounting for 6.70% of the total association). Ye et al. (2025) also found that self-control and perceived stress sequentially mediated the association between physical activity and smartphone addiction. Liu and Sun (2023) showed that physical activity was associated with interpersonal distress through the sequential pathway of “self-control → smartphone addiction” (*β* = −0.012, 95% *CI* [−0.033, −0.003]). Yin et al. (2024) also found that physical activity was associated with sleep quality through the sequential pathway of “self-control → smartphone addiction.” Together, these studies suggest that “physical activity → self-control → health behavior (or reduced addictive behavior)” may be a cross-contextually consistent association pathway; however, none of these studies can establish causality.

The sequential pathway observed in the present study can be understood from two complementary theoretical perspectives, although these explanations are theoretical speculations and were not directly tested in the present study. First, the Strength Model of Self-Control (Baumeister et al., 2007). Physical activity, as a behavior requiring sustained volitional effort, may serve as training for self-control capacity. When individuals persist in completing exercise plans and overcome fatigue and inertia, their self-control resources may be expanded. Second, Self-Determination Theory (Xiang, 2013). When individuals’ basic psychological needs are satisfied through physical activity, their intrinsic motivation may be activated, and health behaviors may gradually be internalized from external constraints into self-endorsed lifestyle habits (Gilbert et al., 2022). Together, these perspectives suggest a possible psychological process from “capacity training” to “lifestyle internalization”.

In addition, the sequential pathway observed in the present study may be regarded as a preliminary theoretical integration consistent with certain assumptions of the I-PACE model (Brand et al., 2016): physical activity may serve as a “training” input for self-control within executive function; enhanced self-control may reduce individuals’ reliance on smartphones for emotion regulation at the affective-cognitive level; and health-promoting lifestyle may provide alternative sources of satisfaction and positive behavioral choices. At the behavioral execution level, these factors may jointly be associated with a lower risk of addiction. However, this integration still requires validation in longitudinal and experimental studies.

In summary, the present study identified a pattern of associations consistent with a sequential mediation model, providing a preliminary theoretical framework for understanding the psychological associations between physical activity and smartphone addiction. Its theoretical implications are threefold: (1) it suggests a possible sequential association between self-control and health-promoting lifestyle, rather than treating them merely as parallel mediators; (2) it provides preliminary empirical clues for integrating the Strength Model of Self-Control and Self-Determination Theory in the context of addictive behaviors; and (3) it is consistent with certain assumptions of the I-PACE model and suggests a possible intervention direction from health behavior to addictive behavior. This framework offers preliminary implications for the prevention and intervention of smartphone addiction among university students: intervention strategies may focus not only on enhancing individuals’ self-control capacity but also on facilitating the translation of this capacity into a health-promoting lifestyle, potentially forming a positive cycle of “capacity enhancement → behavior internalization → addiction resistance.” However, given the cross-sectional design, these intervention implications cannot establish causal mechanisms or intervention efficacy and still require validation in future research.

## 6 Limitations and future directions

### 6.1 Limitations

The following limitations should be considered when interpreting the findings of this study.

First, the cross-sectional design precludes causal inference. This study employed a one-time questionnaire survey, with all variables measured at a single time point. Although structural equation modeling revealed statistical path relationships among the variables, good model fit does not equate to established causality. Specifically, the effects of physical activity on self-control, health-promoting lifestyles, and smartphone addiction may involve cumulative and lagged effects—for example, the shaping of self-control capacity through regular exercise may require weeks or even months of sustained engagement to manifest, a temporal process that a cross-sectional design cannot capture. More importantly, the possibility of reverse causality and reciprocal relationships cannot be ruled out: college students with higher levels of smartphone addiction may tend to be more sedentary and less engaged in physical activity, and the absence of a health-promoting lifestyle could be an antecedent rather than a consequence of diminished self-control. Therefore, the effect sizes reported in this study should be interpreted as statistical descriptions of contemporaneous correlations, rather than as unbiased estimates of causal effects.

Second, the sample source is homogeneous, and external validity has clear boundaries. All participants were recruited from eight universities in Hainan Province, and the sample comprised only freshmen (55.1%) and sophomores (44.9%), with juniors and seniors absent. This sample composition entails two major constraints. On the one hand, Hainan has a tropical monsoon climate with high temperature and humidity throughout the year, resulting in a relatively narrow comfort window for outdoor physical activity. College students’ physical activity patterns—such as preferring morning or evening exercise or engaging in indoor sports—may systematically differ from those in temperate or frigid regions, and climatic conditions may indirectly affect smartphone use by influencing the time available for outdoor activities. On the other hand, lower-grade students have not yet encountered the stressors specific to upper-grade students, such as graduation thesis writing, job competition, and postgraduate entrance examinations; their smartphone use patterns (likely more for socializing and entertainment) may differ fundamentally from those of senior students (likely more for information seeking and stress coping). Taken together, these factors delimit the direct generalizability of the present conclusions to “lower-grade college students in tropical regions,” and the statistical basis for extending them to the national college student population is insufficient.

Third, the measurement tools rely on self-report, posing risks of methodological bias. All four core variables—physical activity, self-control, health-promoting lifestyles, and smartphone addiction—were measured using self-report scales. Although Harman’s single-factor test was conducted (with the common method variance accounting for 17.3%, below the 40% threshold), this method can only detect immediate and explicit biases; it cannot exclude the influence of implicit biases such as social desirability effects (e.g., underreporting smartphone usage time), recall bias (e.g., overestimating exercise frequency over the past week), and anchoring effects. In particular, for self-reported physical activity, research indicates that individuals tend to systematically overestimate their own activity levels by approximately 30 to 50%, a systematic error that may affect the precision of the effect size estimates.

Fourth, the model has limited explanatory power, and the issue of omitted variables exists. The proposed model explained 21.9% of the variance in smartphone addiction (*R^2^* = 0.219), meaning that about 78% of the variance remains unaccounted for. In other words, although all four pathways examined in this study reached statistical significance, in terms of effect magnitude they explain only about one-fifth of the individual differences in smartphone addiction. The model may have omitted other important mediating variables (e.g., psychological resilience, perceived stress, social anxiety, subjective well-being) or moderating variables (e.g., gender, personality traits, family socioeconomic status), the inclusion of which could alter the estimates of the current path coefficients or even reveal more complex interactive effects.

Fifth, the differential effects of specific dimensions of physical activity were not examined. Physical activity was treated as a global construct using a brief scale that assessed overall activity level, without distinguishing the specific effects of intensity (low/moderate/vigorous), frequency (sessions per week), duration (minutes per session), or type of exercise (aerobic/anaerobic/team/individual). While this approach enhances model parsimony, it comes at the cost of not addressing practically relevant questions, such as: “What frequency, duration, and type of exercise are most effective in reducing smartphone addiction?”—precisely the dose–response information needed for intervention design.

Sixth, there is a limitation in the operationalization of health-promoting lifestyles. Health-promoting lifestyles were incorporated into the model as a global multidimensional construct. Although this approach revealed its overall mediating effect, it does not allow identification of which specific dimension (e.g., nutrition, exercise, stress management, interpersonal relationships) plays a key role in the chain pathway. Different dimensions may have distinct mediating mechanisms, and aggregating them into a single construct may obscure these important differential effects.

### 6.2 Future directions

Based on the six limitations outlined above, future research may advance the field along the following lines.

First, longitudinal tracking and cross-lagged designs should be adopted. It is recommended that future studies conduct longitudinal surveys with at least three time points, spaced 3 to 6 months apart, and employ cross-lagged panel models (CLPM) or random-intercept cross-lagged panel models (RI-CLPM) to examine the dynamic causal relationships among variables. Such designs can effectively distinguish between-group differences from within-person changes, more accurately estimate the lagged effects of physical activity on smartphone addiction, and clarify the temporal ordering of mediating variables, thereby providing stronger temporal logic for causal inference.

Second, the sample source should be expanded to enable cross-climatic and cross-grade comparisons. Sampling should be extended to diverse climatic regions—including temperate, frigid, and plateau zones—and encompass various types of higher education institutions, such as comprehensive universities, normal universities, science and engineering universities, and vocational colleges. Senior (third- and fourth-year) undergraduate students as well as graduate students should also be included. More importantly, future research should design a dedicated cross-climatic comparative framework to test whether temperature and humidity indirectly moderate the mediating pathways of this study by influencing willingness to engage in physical activity. If such moderating effects are confirmed, the model parameters estimated in the present study should be understood as context-specific estimates under particular climatic conditions rather than as cross-contextually invariant constants. Cross-cultural comparisons (e.g., China vs. Southeast Asian countries vs. Western countries) would also help test the robustness of the present model across different educational and cultural backgrounds.

Third, multi-modal objective measurement tools should be introduced. Wrist-worn accelerometers (e.g., ActiGraph) or smart bracelets could be used to continuously record physical activity over 7 consecutive days, allowing precise calculation of activity intensity and energy expenditure in metabolic equivalents (METs). Smartphone use duration, unlock frequency, and application type distribution could be automatically tracked using monitoring applications such as iOS Screen Time or Android Digital Wellbeing, replacing self-report assessments of smartphone use behaviors. In addition, self-control could be supplemented by behavioral experiments (e.g., Stroop tasks, delay-discounting tasks) to reduce the subjective bias inherent in self-report scales.

Fourth, the theoretical model should be refined by exploring multiple mediation and moderation effects. It is suggested that variables such as psychological resilience, perceived stress, social anxiety, and subjective well-being be incorporated into extended models to test whether they exert additional mediating or moderating roles between physical activity and smartphone addiction. Furthermore, machine learning-based importance ranking methods (e.g., random forest feature importance evaluation) could be employed to identify the core factors that contribute most to predicting smartphone addiction among a wide array of candidate variables, providing data-driven evidence for streamlining and optimizing the theoretical model.

Fifth, the dimensions of physical activity should be disaggregated to explore dose–response relationships. Future studies should adopt multi-dimensional physical activity measures (e.g., the long form of the International Physical Activity Questionnaire, IPAQ-L) to separately examine the differential effects of exercise intensity (light/moderate/vigorous), frequency (days per week), duration (minutes per session), and type of activity (aerobic/anaerobic/team/individual/outdoor) on smartphone addiction. Isotemporal substitution models could be applied to estimate the marginal effects of replacing sedentary time with physical activity of varying intensities on smartphone addiction, thereby providing empirical foundations for developing precise exercise prescriptions.

Sixth, randomized controlled trial (RCT) intervention studies should be conducted. The most direct approach to establishing causality remains experimental manipulation. Future research should design an 8- to 12-week structured exercise intervention RCT, with an exercise intervention group and a wait-list control group, and track the trajectories of self-control, health-promoting lifestyles, and smartphone addiction before and after the intervention. Mediation analyses could then be used to test whether the chain mechanism proposed in this study holds in the intervention context. Such studies would not only provide causal evidence but also offer directly actionable guidance for university student affairs departments in developing evidence-based intervention protocols.

## 7 Conclusion

Drawing upon self-determination theory and the strength model of self-control, this study constructed and tested a chain mediation model of “physical activity → self-control → health-promoting lifestyle → smartphone addiction.” The model demonstrated good overall fit (CFI = 0.990, RMSEA = 0.039, SRMR = 0.035), with all path coefficients reaching statistical significance (*p* < 0.05). Based on this model, the following findings were obtained:

1. Direct effect: Physical activity had a statistically significant direct negative predictive effect on smartphone addiction among college students (direct effect *β* = −0.249, accounting for 61.03% of the total effect). This result supports the statistical hypothesis that physical activity can be considered an independent correlational factor for smartphone addiction.
2. Mediating effect of self-control: Self-control played a partial mediating role between physical activity and smartphone addiction (indirect effect *β* = −0.090, accounting for 22.06% of the total effect). This pathway suggests that college students with higher levels of physical activity tend to report greater self-control capacity, and that enhanced self-control is statistically associated with lower levels of smartphone addiction.
3. Mediating effect of health-promoting lifestyle: Health-promoting lifestyle also mediated the relationship (indirect effect *β* = −0.033, accounting for 8.09% of the total effect). This pathway suggests that college students who engage in more physical activity are more inclined to adopt a systematic health-promoting lifestyle, and such a healthy lifestyle is statistically associated with lower smartphone addiction.
4. Chain mediating effect: This study incorporated health-promoting lifestyle as a global multidimensional construct into the chain model, and the data supported the chain pathway of “physical activity → self-control → health-promoting lifestyle → smartphone addiction” (chain mediating effect *β* = −0.036, accounting for 8.82% of the total effect, 95% *CI* [−0.060, −0.014]). Although statistically significant, this effect size was relatively small (less than 10% of the total effect), and its practical significance for clinical or educational interventions warrants cautious interpretation.

### 7.1 Practical implications

From a researcher’s standpoint, the implications of these findings are best understood as empirically grounded hypotheses that point toward promising directions for future investigation, rather than as ready-to-implement policy prescriptions. The following reflections are offered with full acknowledgment of the cross-sectional nature of our data and the specificity of our sample (*N* = 712 college students from eight universities in Hainan Province). All interpretations should be read with the understanding that statistical associations do not warrant causal claims, and any translational applications must await experimental validation.

1. Reframing physical activity’s role in digital wellbeing. The direct path from physical activity to smartphone addiction (*β* = −0.179, accounting for 61.03% of the total effect) represents the most substantial portion of the observed association. For researchers and practitioners in university settings, this finding directs attention to campus physical activity environments as potentially relevant contexts for health promotion. However, what we observe is a correlational pattern, not a causal mechanism. The question that merits priority in subsequent research is whether systematically increasing physical activity engagement produces detectable changes in problematic smartphone use. Until such evidence emerges from well-designed intervention studies, the prudent position is to regard physical activity as one element within multifactorial health frameworks, rather than as a targeted “intervention” for smartphone addiction per se.
2. Examining self-control as a behavioral pathway. Our mediation analysis revealed that self-control transmits approximately 22.06% of the total effect, suggesting that the physical activity–smartphone addiction association may operate, in part, through self-regulatory capacities. This finding offers a conceptually grounded rationale for designing physical education curricula that intentionally incorporate self-regulation components—goal-setting exercises, self-monitoring protocols, and delayed gratification tasks—as a means of addressing both physical and psychological outcomes simultaneously. Yet it is essential to stress that our cross-sectional mediation model cannot establish the temporal sequence implied by this pathway. Whether physical activity actually strengthens self-control, or whether individuals with higher baseline self-control are simply more likely to engage in physical activity (and less prone to smartphone addiction), remains an open empirical question. Randomized controlled trials are indispensable for adjudicating between these competing explanations.
3. The modest contribution of health-promoting lifestyle. The chain mediation pathway through self-control and health-promoting lifestyle yielded a statistically significant but quantitatively small effect (*β* = −0.034, 95% CI [−0.057, −0.013]), representing only 8.82% of the total effect. From a research perspective, this modest magnitude warrants careful interpretation: statistical significance should not be conflated with practical or clinical significance. If this pathway withstands replication in longitudinal designs, its contribution would likely be supplementary rather than primary. For those designing future studies, this suggests that holistic lifestyle interventions—incorporating sleep hygiene, nutritional balance, and stress management—may offer incremental benefits, but should not overshadow the more substantial direct and self-control-mediated pathways that emerged in our data.
4. Testing the integrated model hypothesis. The sequential mechanism we observed—physical activity → self-control → health-promoting lifestyle → reduced smartphone addiction—finds theoretical coherence within the Strength Model of Self-Control and Self-Determination Theory. As a hypothesis-generating finding, it proposes that physical activity may serve as a “capacity builder” that cascades through cognitive and behavioral layers to influence digital wellbeing. However, we must be forthright about a fundamental limitation: chain mediation from cross-sectional data cannot substantiate the directional flow we have modeled. Alternative ordering—for instance, that health-promoting lifestyle enables better self-control, which in turn facilitates physical activity engagement—is equally plausible given our data. The integrated intervention approach (combining physical activity, self-control training, and lifestyle modification) that follows logically from our model should therefore be approached as a testable theoretical proposition, not as an established protocol. Longitudinal tracking studies and dismantling designs in intervention trials would be required to validate both the causal architecture and the practical efficacy of such integrated programs.
5. Addressing generalizability constraints. Our sample—drawn from eight universities within a single tropical province of China (Hainan), and restricted to freshmen and sophomores—inevitably raises questions about the external validity of our findings. Several contextual factors warrant particular attention. First, Hainan’s tropical climate, characterized by high temperature and humidity throughout the year, may systematically shape students’ physical activity patterns in ways that differ from those in temperate or cold regions. Second, the exclusion of upperclassmen is consequential: juniors and seniors face distinct pressures—including job-seeking and postgraduate entrance examinations—that may engender qualitatively different patterns of smartphone use compared with lower-grade students. Third, university populations in other geographic regions, urban versus rural institutional settings, or cultural contexts with different norms around technology use may well exhibit different patterns of association. Moreover, the cross-sectional design precludes any claims about temporal precedence. Collectively, these limitations define clear priorities for the research agenda: replication across diverse samples, particularly in varied geographic and cultural contexts and across all undergraduate years; longitudinal cohort studies to clarify temporal ordering; and pre-registered pilot trials with objective behavioral outcome measures (e.g., actual screen time data, not merely self-reported usage) to translate correlational findings into causally credible evidence. Without such programmatic work, the practical implications discussed here remain at the level of research-informed speculation.

In sum, we view these findings as contributing a theoretically grounded and empirically testable set of hypotheses to the growing literature on physical activity, self-regulation, and digital health. The most immediate contribution of this study, in our view, lies not in its policy implications but in its capacity to guide the design of future studies that can address the causal and generalizability questions we have raised. We hope that this work encourages other researchers to interrogate these pathways using stronger inferential designs, and we look forward to seeing whether the pattern we observed holds under more rigorous empirical scrutiny.

## Data Availability

The original contributions presented in the study are included in the article/Supplementary material, further inquiries can be directed to the corresponding author.

## References

[ref1] Angius L. Santarnecchi E. Pascual-Leone A. Marcora S. M. (2019). Transcranial direct current stimulation over the left dorsolateral prefrontal cortex improves inhibitory control and endurance performance in healthy individuals. Neuroscience 419, 34–45. doi: 10.1016/j.neuroscience.2019.08.052, 31493549

[ref2] Audiffren M. André N. (2015). The strength model of self-control revisited: linking acute and chronic effects of exercise on executive functions. J. Sport Health Sci. 4, 30–46. doi: 10.1016/j.jshs.2014.09.002

[ref3] Baumeister R. F. Bratslavsky E. Muraven M. Tice D. M. (1998). Ego depletion: is the active self a limited resource? J. Pers. Soc. Psychol. 74, 5, 1252–1265. doi: 10.1037/0022-3514.74.5.1252, 9599441

[ref4] Baumeister R. F. Vohs K. D. Tice D. M. (2007). The strength model of self-control. Curr. Dir. Psychol. Sci. 16, 351–355. doi: 10.1111/j.1467-8721.2007.00534.x

[ref5] Boat R. Cooper S. B. (2019). Self-control and exercise: a review of the bi-directional relationship. Brain plasticity (Amsterdam, Netherlands) 5, 97–104. doi: 10.3233/bpl-190082, 31970063 PMC6971834

[ref6] Brand M. Young K. S. Laier C. Wölfling K. Potenza M. N. (2016). Integrating psychological and neurobiological considerations regarding the development and maintenance of specific internet-use disorders: an interaction of person-affect-cognition-execution (I-PACE) model. Neurosci. Biobehav. Rev. 71, 252–266. doi: 10.1016/j.neubiorev.2016.08.033, 27590829

[ref7] Byun K. Hyodo K. Suwabe K. Ochi G. Sakairi Y. Kato M. . (2014). Positive effect of acute mild exercise on executive function via arousal-related prefrontal activations: an fNIRS study. NeuroImage 98, 336–345. doi: 10.1016/j.neuroimage.2014.04.067, 24799137

[ref8] Cao W. Guo Y. Peng W. Zhen J. (2016). Development and psychometric tests of a Chinese version of the HPLP-II scales. Chin. J. Dis. Control Prev. 20, 286–289. doi: 10.16462/j.cnki.zhjbkz.2016.03.018

[ref9] Cassilhas R. C. Tufik S. d Mello M. T. (2016). Physical exercise, neuroplasticity, spatial learning and memory. Cell. Mol. Life Sci. 73, 975–983. doi: 10.1007/s00018-015-2102-0 PMC11108521 26646070

[ref10] CNNIC (2026). The 57th Statistical Report on Internet Development in China https://www.cnnic.cn/n4/2026/0304/c88-11549.html

[ref11] Colcombe S. J. Erickson K. I. Scalf P. E. Kim J. S. Prakash R. McAuley E. . (2006). Aerobic exercise training increases brain volume in aging humans. J. Gerontol. A Biol. Sci. Med. Sci. 61, 1166–1170. doi: 10.1093/gerona/61.11.1166, 17167157

[ref12] Davis C. L. Tomporowski P. D. McDowell J. E. Austin B. P. Miller P. H. Yanasak N. E. . (2011). Exercise improves executive function and achievement and alters brain activation in overweight children: a randomized, controlled trial. Health Psychol. 30, 91–98. doi: 10.1037/a0021766, 21299297 PMC3057917

[ref13] Ding Z. Pei Q. Fang T. Xiao L. Yin D. Sun Z. (2025). Assessment of smartphone addiction and its relationship with health-promoting lifestyle profile and intuitive eating behavior: a cross-sectional study. Front. Psych. 16:1682921. doi: 10.3389/fpsyt.2025.1682921, 41113199 PMC12531270

[ref14] Englert C. (2016). The strength model of self-control in sport and exercise psychology. Front. Psychol. 7:314. doi: 10.3389/fpsyg.2016.00314, 26973590 PMC4773600

[ref15] Erickson K. I. Voss M. W. Prakash R. S. Basak C. Szabo A. Chaddock L. . (2011). Exercise training increases size of hippocampus and improves memory. Proc. Natl. Acad. Sci. USA 108, 3017–3022. doi: 10.1073/pnas.1015950108, 21282661 PMC3041121

[ref16] Feng J. Yan Y. Lu Y. Xu J. Sun B. Feng L. (2019). Progress in research of exercise intervention for drug detoxification. China Sport Sci. Technol. 55, 3–11. doi: 10.16470/j.csst.2019626

[ref17] Fornell C. Larcker D. F. (1981). Evaluating structural equation models with unobservable variables and measurement error. J. Mark. Res. 18, 39–50. doi: 10.1177/002224378101800104

[ref18] Gilbert W. Bureau J. S. Poellhuber B. Guay F. (2022). Educational contexts that nurture students' psychological needs predict low distress and healthy lifestyle through facilitated self-control. Curr. Psychol. 42, 29661–29681. doi: 10.1007/s12144-022-04019-5, 36468166 PMC9684939

[ref19] Gong W. Zhao H. Wei Z. Feng T. Feng P. (2025). The functional connectivity between the dorsolateral prefrontal cortex and the medial prefrontal cortex underlying the association between self-control and delay discounting. Brain Struct. Funct. 230:71. doi: 10.1007/s00429-025-02936-2, 40397223

[ref20] Grasdalsmoen M. Eriksen H. R. Lønning K. J. Sivertsen B. (2020). Physical exercise, mental health problems, and suicide attempts in university students. BMC Psychiatry 20:175. doi: 10.1186/s12888-020-02583-3, 32299418 PMC7164166

[ref21] Grimaldi-Puyana M. Fernández-Batanero J. M. Fennell C. Sañudo B. (2020). Associations of objectively-assessed smartphone use with physical activity, sedentary behavior, mood, and sleep quality in young adults: a cross-sectional study. Int. J. Environ. Res. Public Health 17:3499. doi: 10.3390/ijerph17103499, 32429550 PMC7277080

[ref22] Guo K.-l. Ma Q.-s. Yao S.-j. Chao L. Hui Z. Jiang J. . (2022). The relationship between physical exercise and mobile phone addiction tendency of university students in China: a moderated mediation model [original research]. Front. Psychol. 13–2022. doi: 10.3389/fpsyg.2022.730886 PMC8884265 35237204

[ref23] Hare T. A. Camerer C. F. Rangel A. (2009). Self-control in decision-making involves modulation of the vmPFC valuation system. Science 324, 646–648. doi: 10.1126/science.1168450, 19407204

[ref24] Hashimoto K. (1990). Stress, Exercise and Quality of life Proceedings. Beijing Asian Games a Scientific Congress. Zhejiang Zhimei Printing & Packaging Co., Ltd.

[ref25] Hayes A. F. (2017). Introduction to Mediation, Moderation, and Conditional process Analysis: A Regression-based Approach. Guilford publications.

[ref26] Hofmann W. Friese M. Strack F. (2009). Impulse and self-control from a dual-systems perspective. Perspect. Psychol. Sci. 4, 162–176. doi: 10.1111/j.1745-6924.2009.01116.x, 26158943

[ref27] Hu L. t. Bentler P. M. (1999). Cutoff criteria for fit indexes in covariance structure analysis: conventional criteria versus new alternatives. Struct. Equ. Model. 6, 1–55. doi: 10.1080/10705519909540118

[ref28] Hu Y. Yang Z. Zhou X. (2026). Smartphone dependence and sport participation among Chinese adolescents: chain mediation by self-control and health beliefs and direct-path moderation by interpersonal support. Front. Psychol. 17, 1876296–1876296. doi: 10.3389/fpsyg.2026.1876296, 42453455 PMC13368333

[ref29] Hu M. Zeng N. Gu Z. Zheng Y. Xu K. Xue L. . (2021). Short-term high-intensity interval exercise promotes motor cortex plasticity and executive function in sedentary females. Front. Hum. Neurosci. 15, 620958–620958. doi: 10.3389/fnhum.2021.620958, 33967719 PMC8102987

[ref30] Jiang Z. Zhao X. (2016). Self-control and problematic mobile phone use in Chinese college students: the mediating role of mobile phone use patterns. BMC Psychiatry 16:416. doi: 10.1186/s12888-016-1131-z, 27876032 PMC5120559

[ref31] Kardefelt-Winther D. (2014). A conceptual and methodological critique of internet addiction research: towards a model of compensatory internet use. Comput. Hum. Behav. 31, 351–354. doi: 10.1016/j.chb.2013.10.059

[ref32] Ke Y. Liu X. Xu X. He B. Wang J. Zuo L. . (2024). Self-esteem mediates the relationship between physical activity and smartphone addiction of Chinese college students: a cross-sectional study. Front. Psychol. 14, 1256743–1256743. doi: 10.3389/fpsyg.2023.1256743, 38250119 PMC10797096

[ref33] Kline R. B. (2016). Principles and Practice of Structural Equation Modeling, vol. 10. 4e éd Edn New York: Guilford.

[ref34] Kline R. B. (2023). Principles and Practice of Structural Equation Modeling. Guilford publications.

[ref35] Kushlev K. Leitao M. R. (2020). The effects of smartphones on well-being: theoretical integration and research agenda. Curr. Opin. Psychol. 36, 77–82. doi: 10.1016/j.copsyc.2020.05.001 32563707

[ref36] Lai C. Cai P. Liao J. Li X. Wang Y. Wang M. . (2025). Exploring the relationship between physical activity and smartphone addiction among college students in Western China. Front. Public Health 13, 1530947–1530947. doi: 10.3389/fpubh.2025.1530947, 40061470 PMC11885492

[ref37] Li X. Chen W. lI Z. Wang R. (2023). Effect of physical exercise on mobile phone addiction among college students: the mediating role of psychological distress. China J. Health Psychol. 31, 423–428. doi: 10.13342/j.cnki.cjhp.2023.03.020

[ref38] Li Y. Li G. Liu L. Wu H. (2020). Correlations between mobile phone addiction and anxiety, depression, impulsivity, and poor sleep quality among college students: a systematic review and meta-analysis. J. Behav. Addict. 9, 551–571. doi: 10.1556/2006.2020.00057, 32903205 PMC8943681

[ref39] Liang D. (1994). Stress levels among college students and their relationship with physical exercise. Chin. Ment. Health J.(1), 5–6. Available online at: https://kns.cnki.net/kcms2/article/abstract?v=35M_ufc67ztqXVtLoHbMZH4v1BLqe2LMccP0aCO1XuoEAmBMGyUJn3Cwqwy5OawnPv2kn0g3wa-Uzay_jQEP5hFV-_yxYmgdeXmKwSN56YhPjjjc6xg73AHEh9jtVKMEi6OalBCkMw-MOn0w3aQq_foAapF_E7ynz1HuKo27eJkomIKFJBPPfg==&uniplatform=NZKPT&language=CHS

[ref40] Lin H. Fan H. Fu Q. Li S. Liu Q. (2025). The mediating role of self-control between physical activity and mobile phone addiction in adolescents: a meta-analytic structural equation modeling approach. Front. Psych. 16, 1446872–1446872. doi: 10.3389/fpsyt.2025.1446872, 40458777 PMC12127393

[ref41] Liu C. Sun Z. (2023). The relationship between physical activity and interpersonal distress in college students: the chain mediating role of self-control and mobile phone addiction. Psicol. Reflex Crit. 36, 18–18. doi: 10.1186/s41155-023-00261-3, 37530865 PMC10397154

[ref42] Long J. Liu T.-Q. Liao Y.-H. Qi C. He H.-Y. Chen S.-B. . (2016). Prevalence and correlates of problematic smartphone use in a large random sample of Chinese undergraduates. BMC Psychiatry 16:408. doi: 10.1186/s12888-016-1083-3, 27855666 PMC5114822

[ref43] Morgan W. P. (1985). Affective beneficence of vigorous physical activity. Med. Sci. Sports Exerc. 17, 94–100. doi: 10.1249/00005768-198502000-00015 3157040

[ref44] Muraven M. Baumeister R. F. (2000). Self-regulation and depletion of limited resources: does self-control resemble a muscle? Psychol. Bull. 126, 247–259. doi: 10.1037/0033-2909.126.2.247, 10748642

[ref45] Muraven M. Baumeister R. F. Tice D. M. (1999). Longitudinal improvement of self-regulation through practice: building self-control strength through repeated exercise. J. Soc. Psychol. 139, 446–457. doi: 10.1080/00224549909598404, 10457761

[ref46] Podsakoff P. M. MacKenzie S. B. Lee J.-Y. Podsakoff N. P. (2003). Common method biases in behavioral research: a critical review of the literature and recommended remedies. J. Appl. Psychol. 88, 879–903. doi: 10.1037/0021-9010.88.5.879, 14516251

[ref47] Preacher K. J. Hayes A. F. (2008). Asymptotic and resampling strategies for assessing and comparing indirect effects in multiple mediator models. Behav. Res. Methods 40, 879–891. doi: 10.3758/BRM.40.3.879, 18697684

[ref48] Qi J. Zhou X. Li J. Liu B. (2025). The impact of regular physical exercise on mobile phone addiction tendency among Chinese college students: the mediating role of trait boredom and the moderating role of self-control [original research]. Front. Psychol. 16–2025. doi: 10.3389/fpsyg.2025.1671836 PMC12580359 41190115

[ref49] Ryan R. M. Deci E. L. (2000). Self-determination theory and the facilitation of intrinsic motivation, social development, and well-being. Am. Psychol. 55, 68–78. doi: 10.1037/0003-066X.55.1.68, 11392867

[ref50] Standage M. Gillison F. B. Ntoumanis N. Treasure D. C. (2012). Predicting students' physical activity and health-related well-being: a prospective cross-domain investigation of motivation across school physical education and exercise settings. J. Sport Exerc. Psychol. 34, 37–60. doi: 10.1123/jsep.34.1.37, 22356882

[ref51] Tan S. Guo Y. (2008). Revision of self-control scale for Chinese college students. Chin. J. Clin. Psychol. 5, 468–470. doi: 10.16128/j.cnki.1005-3611.2008.05.022

[ref52] Thapliyal V. Kushwaha J. Thakur G. S. (2025). Effect of ten-week yoga intervention on problematic smartphone usage in university students: a randomized controlled trial. J. Ayurv. Integ. Med. 16:101199. doi: 10.1016/j.Jaim.2025.101199, 40957177 PMC12466232

[ref53] Voss M. W. Heo S. Prakash R. S. Erickson K. I. Alves H. Chaddock L. . (2013). The influence of aerobic fitness on cerebral white matter integrity and cognitive function in older adults: results of a one-year exercise intervention. Hum. Brain Mapp. 34, 2972–2985. doi: 10.1002/hbm.22119, 22674729 PMC4096122

[ref54] Walker S. N. Sechrist K. R. Pender N. J. (1987). The health-promoting lifestyle profile: development and psychometric characteristics. Nurs. Res. 36, 76–81. doi: 10.1097/00006199-198703000-00002, 3644262

[ref55] Wang F. (2025). Effects of physical activity on mobile phone addiction among university students: the mediating roles of self-control and resilience. Front. Psychol. 16:1503607. doi: 10.3389/fpsyg.2025.1503607, 40231003 PMC11994967

[ref56] Wang Q. Chen Y. Li L. (2024). Effects of physical activity and self-control on mobile phone addiction in college students: a cross-lagged study in China. Front. Psychol. 15, 1417379–1417379. doi: 10.3389/fpsyg.2024.1417379, 39100561 PMC11297356

[ref57] Won M.-h. Sin S.-h. (2018). Mediating effect of smartphone addiction predisposition on the relationship between perceived stress and health-promoting lifestyle in university students. J. Korean Converg. Soc. 9, 449–458.

[ref58] Xiang M. (2013). The path of promotion on teenagers' physical exercise and health-related well-being: based on self-determination theory model. China Sport Sci. 33, 21–28. doi: 10.16469/j.css.2013.08.009

[ref59] Xiao T. Jiao C. Yao J. Yang L. Zhang Y. Liu S. . (2021). Effects of basketball and Baduanjin exercise interventions on problematic smartphone use and mental health among college students: a randomized controlled trial. Evid. Based Complement. Alternat. Med. 2021, 1–12. doi: 10.1155/2021/8880716, 33574886 PMC7864751

[ref60] Xiao W. Wu J. Yip J. Shi Q. Peng L. Lei Q. E. . (2022). The relationship between physical activity and Mobile phone addiction among adolescents and Young adults: systematic review and Meta-analysis of observational studies. JMIR Public Health Surveill. 8, e41606–e41606. doi: 10.2196/41606, 36515994 PMC9798266

[ref61] Xie Y. Pei Q. Chen Y. Xiao L. Yin D. (2026). The influence of smartphone addiction on sleep quality among college students: the parallel mediating roles of perceived stress and health-promoting lifestyle. PLoS One 21:e0340852. doi: 10.1371/journal.Pone.0340852, 41678505 PMC12900322

[ref62] Xiong Z. Zhao H. Xu T. Huang J. Yang C. Feng T. . (2025). Resting-state functional connectivity insights: dorsolateral prefrontal cortex and superior parietal lobule in self-control and cognitive reappraisal. Brain Cogn. 190:106361. doi: 10.1016/j.Bandc.2025.106361, 41005016

[ref63] Xiong J. Zhou Z. Chen W. You Z. Zhai Z. (2012). Development of the mobile phone addiction tendency scale for college students. Chin. Ment. Health J., 26, 222–225. Available online at: https://kns.cnki.net/kcms2/article/abstract?v=hiwAvoCB7A04dP2mAnUIMg6_uuqMVjhIJwD3HsiY4zQPvit1-yEpNstNXAu_jG7vOSkCKO_N8u13FSCbr2Sbw0WwEy_dwypXotTwUVOotALgBsWSxPzCZPjhREKFQ8iKEnzIeyCHW2oOr9HCN-iNVVS1ZeQyLBT80teCglGwHxY_PRXFAu6iMw==&uniplatform=NZKPT&language=CHS

[ref64] Yang G. Tan G. X. Li Y. X. Liu H. Y. Wang S. T. (2019). Physical exercise decreases the Mobile phone dependence of university students in China: the mediating role of self-control. Int. J. Environ. Res. Public Health 16, 4098–4098. doi: 10.3390/ijerph16214098, 31652978 PMC6862431

[ref65] Yang G. Li Y. Liu S. Liu C. Jia C. Wang S. (2021). Physical activity influences the mobile phone addiction among Chinese undergraduates: the moderating effect of exercise type. J. Behav. Addict. 10, 799–810. doi: 10.1556/2006.2021.00059, 34546969 PMC8997213

[ref66] Yang C. Zeng G. (2017). The impact of tai chi practice on internet addiction among college students. Chin. J. Sch. Health 38, 292–294. doi: 10.16835/j.cnki.1000-9817.2017.02.041

[ref67] Ye Z. Zhang T. Peng Y. Rao W. Jia P. (2025). Effects of physical activity on smartphone addiction in Chinese college students-chain mediation of self-control and stress perception. BMC Public Health 25, 1532–1532. doi: 10.1186/s12889-025-22720-5, 40275263 PMC12020245

[ref68] Yin Z. Yang C. Liu T. Yu J. Yu X. Huang S. . (2024). The relationship between physical activity and sleep quality among college students: the chain-mediating effects of self-control and mobile phone addiction. PLoS One 19:e0315930. doi: 10.1371/journal.Pone.0315930, 39700194 PMC11658481

[ref69] Yu L. Chen Z. Huang X. Tao X. Lv Y. (2025). The relationship between physical activity and Mobile phone addiction in college students: a systematic review and Meta-analysis. Behav. Sci. 15, 1325–1325. doi: 10.3390/bs15101325, 41153116 PMC12561036

[ref70] Zhong W. Wang Y. Zhang G. (2020). The impact of physical activity on college students’ mobile phone dependence: the mediating role of self-control. Int. J. Ment. Health Addict. 19, 1–16. doi: 10.1007/s11469-020-00308-x

