# Supplementary material for: The effect of physical activity on smartphone addiction among Chinese college students: a chain mediation of self-control and health-promoting lifestyle
Source: Front Psychol. 2026 Oct 19;17:1990964. doi: 10.3389/fpsyg.2026.1990964 (PMC13724419; doi:10.3389/fpsyg.2026.1990964)
Supplement: Supplementary file 1 [file Supplementary_file_1.docx]

Supplementary materials:

| Table S1 Normality Test for All Observed Indicators (N = 712) | | | | | | | | | | | | | | | | | | |
| --- | --- | --- | --- | --- | --- | --- | --- | --- | --- | --- | --- | --- | --- | --- | --- | --- | --- | --- |
|  | Exercise duration | Exercise intensity | Exercise frequency | Withdrawal symptoms | Salience | Social comfort | Mood change | Impulse control | Healthy habits | Resistance to temptation | Focused work | Moderation in entertainment | Interpersonal relationships | Nutrition | Health responsibility | Physical activity (HPL dimension) | Stress management | Spiritual growth |
| Mean | 3.57 | 3.50 | 3.49 | 2.97 | 3.08 | 3.01 | 3.02 | 3.61 | 3.76 | 3.60 | 3.70 | 3.58 | 3.58 | 3.61 | 3.55 | 3.52 | 3.77 | 3.61 |
| SD | 1.05 | 1.18 | 1.16 | 0.71 | 0.77 | 0.75 | 0.74 | 0.68 | 0.73 | 0.70 | 0.75 | 0.75 | 0.62 | 0.66 | 0.61 | 0.62 | 0.72 | 0.66 |
| Skewness | -0.59 | -0.42 | -0.45 | 0.19 | 0.05 | 0.03 | 0.16 | -0.33 | -0.51 | -0.19 | -0.38 | -0.20 | -0.16 | -0.07 | -0.26 | -0.11 | -0.36 | -0.26 |
| S.E. of Skewness | 0.09 | 0.09 | 0.09 | 0.09 | 0.09 | 0.09 | 0.09 | 0.09 | 0.09 | 0.09 | 0.09 | 0.09 | 0.09 | 0.09 | 0.09 | 0.09 | 0.09 | 0.09 |
| Kurtosis | -0.27 | -0.69 | -0.59 | -0.23 | -0.24 | -0.34 | -0.34 | -0.16 | -0.15 | -0.34 | -0.49 | -0.56 | -0.18 | -0.69 | 0.02 | -0.09 | -0.54 | -0.45 |
| S.E. of Kurtosis | 0.18 | 0.18 | 0.18 | 0.18 | 0.18 | 0.18 | 0.18 | 0.18 | 0.18 | 0.18 | 0.18 | 0.18 | 0.18 | 0.18 | 0.18 | 0.18 | 0.18 | 0.18 |

| Table S2 Pearson Correlation Matrix Among All Observed Indicators (N = 712) | | | | | | | | | | | | | | | | | | |
| --- | --- | --- | --- | --- | --- | --- | --- | --- | --- | --- | --- | --- | --- | --- | --- | --- | --- | --- |
|  | Exercise duration | Exercise intensity | Exercise frequency | Withdrawal symptoms | Salience | Social comfort | Mood change | Impulse control | Healthy habits | Resistance to temptation | Focused work | Moderation in entertainment | Interpersonal relationships | Nutrition | Health responsibility | Physical activity (HPL dimension) | Stress management | Spiritual growth |
| Exercise duration | 1 |  |  |  |  |  |  |  |  |  |  |  |  |  |  |  |  |  |
| Exercise intensity | 0.722  ** | 1 |  |  |  |  |  |  |  |  |  |  |  |  |  |  |  |  |
| Exercise frequency | 0.722  ** | 0.784  ** | 1 |  |  |  |  |  |  |  |  |  |  |  |  |  |  |  |
| Withdrawal symptoms | -0.347  ** | -0.334  ** | -0.334  ** | 1 |  |  |  |  |  |  |  |  |  |  |  |  |  |  |
| Salience | -0.348  ** | -0.320  ** | -0.328  ** | 0.878  ** | 1 |  |  |  |  |  |  |  |  |  |  |  |  |  |
| Social comfort | -0.333  ** | -0.305  ** | -0.312  ** | 0.873  ** | 0.851  ** | 1 |  |  |  |  |  |  |  |  |  |  |  |  |
| Mood change | -0.345  ** | -0.320  ** | -0.339  ** | 0.864  ** | 0.844  ** | 0.833  ** | 1 |  |  |  |  |  |  |  |  |  |  |  |
| Impulse control | 0.446  ** | 0.453  ** | 0.454  ** | -0.353  ** | -0.377  ** | -0.329  ** | -0.384  ** | 1 |  |  |  |  |  |  |  |  |  |  |
| Healthy habits | 0.415  ** | 0.432  ** | 0.420  ** | -0.336  ** | -0.336  ** | -0.306  ** | -0.352  ** | 0.842  ** | 1 |  |  |  |  |  |  |  |  |  |
| Resistance to temptation | 0.399  ** | 0.435  ** | 0.435  ** | -0.297  ** | -0.313  ** | -0.273  ** | -0.329  ** | 0.862  ** | 0.832  ** | 1 |  |  |  |  |  |  |  |  |
| Focused work | 0.429  ** | 0.439  ** | 0.446  ** | -0.312  ** | -0.325  ** | -0.293  ** | -0.341  ** | 0.832  ** | 0.852  ** | 0.829  ** | 1 |  |  |  |  |  |  |  |
| Moderation in entertainment | 0.430  ** | 0.441  ** | 0.445  ** | -0.310  ** | -0.309  ** | -0.285  ** | -0.334  ** | 0.853  ** | 0.780  ** | 0.828  ** | 0.797  ** | 1 |  |  |  |  |  |  |
| Interpersonal relationships | 0.326  ** | 0.324  ** | 0.313  ** | -0.293  ** | -0.284  ** | -0.283  ** | -0.314  ** | 0.460  ** | 0.393  ** | 0.432  ** | 0.455  ** | 0.429  ** | 1 |  |  |  |  |  |
| Nutrition | 0.347  ** | 0.361  ** | 0.348  ** | -0.306  ** | -0.309  ** | -0.291  ** | -0.317  ** | 0.477  ** | 0.423  ** | 0.439  ** | 0.466  ** | 0.449  ** | 0.801  ** | 1 |  |  |  |  |
| Health responsibility | 0.364  ** | 0.384  ** | 0.368  ** | -0.297  ** | -0.298  ** | -0.290  ** | -0.319  ** | 0.449  ** | 0.431  ** | 0.444  ** | 0.462  ** | 0.428  ** | 0.775  ** | 0.792  ** | 1 |  |  |  |
| Physical activity (HPL dimension) | 0.316  ** | 0.348  ** | 0.348  ** | -0.301  ** | -0.304  ** | -0.295  ** | -0.317  ** | 0.450  ** | 0.426  ** | 0.444  ** | 0.452  ** | 0.444  ** | 0.782  ** | 0.771  ** | 0.814  ** | 1 |  |  |
| Stress management | 0.330  ** | 0.323  ** | 0.358  ** | -0.225  ** | -0.215  ** | -0.208  ** | -0.263  ** | 0.422  ** | 0.410  ** | 0.415  ** | 0.453  ** | 0.408  ** | 0.715  ** | 0.745  ** | 0.778  ** | 0.772  ** | 1 |  |
| Spiritual growth | 0.309  ** | 0.316  ** | 0.307  ** | -0.300  ** | -0.288  ** | -0.266  ** | -0.301  ** | 0.419** | 0.387  ** | 0.435  ** | 0.420  ** | 0.418  ** | 0.692  ** | 0.738  ** | 0.720  ** | 0.692  ** | 0.691  ** | 1 |
